# Supplementary material for: Allosteric activation of the nitric oxide receptor soluble guanylate cyclase mapped by cryo-electron microscopy
Source: eLife. 2019 Sep 30;8:e50634. doi: 10.7554/eLife.50634 (PMC6839917; doi:10.7554/eLife.50634)
Supplement: Supplementary file 1. [file elife-50634-supp1.docx]

**Supplementary File - Table 1**

**Cryo-EM data acquisition, image processing and model refinement**

| **Imaging/Processing** | **Inactive** | **Active** |
| --- | --- | --- |
| Microscope | Talos Arctica | Talos Arctica |
| Voltage (kV) | 200 | 200 |
| Camera | K3 | K3 |
| Defocus range (µm) | -1.5 to -3.5 | -1.5 to -3.5 |
| Pixel size (Å) | 1.137 | 1.137 |
| Total electron dose (e-/Å^2^) | 60 | 60 |
| Exposure time (s) | 6 | 6 |
| Number of movies | 2,841 | 9,330 |
| Number of frames/movie | 60 | 60 |
| Initial particle number | 675,956 |  |
| Final particle number | 25,828 | 40,469 |
| FSC 0.143 (unmasked/masked, Å) | 8.3 / 5.1 | 7.6 / 5.8 |
|  |  |  |
| **Refinement** |  |  |
| Homology Models (PDB) | α/β H-NOX (2O0C), α/β PAS (4GJ4), α/β CC (3HLS), α CAT (3UVJ), β CAT (2WZ1) | |
| Cα Residues | 1129 | 953 |
| Ligands | Heme | Heme |
|  |  |  |
| **RMSDs** |  |  |
| Bond lengths (Å) | 0.006 | 0.009 |
| Bond angles (˚) | 1.096 | 1.229 |
|  |  |  |
| **Ramachandran** |  |  |
| Favored (%) | 82.57 | 76.35 |
| Allowed (%) | 17.25 | 23.23 |
| Outlier (%) | 0.09 | 0.42 |
| **All Atom Clash score** | 1.38 | 6.92 |
| **Molprobity score** | 1.57 | 2.16 |
| **Accession codes** |  |  |
| EMDB | EMD-20282 | EMD-20283 |
| PDB | PDB - 6PAS | PDB – 6PAT |
