# Supplementary material for: Allosteric activation of the nitric oxide receptor soluble guanylate cyclase mapped by cryo-electron microscopy
Source: eLife. 2019 Sep 30;8:e50634. doi: 10.7554/eLife.50634 (PMC6839917; doi:10.7554/eLife.50634)
Supplement: Supplementary file 2. [file elife-50634-supp2.docx]

Supplemental File - Table 2

**SEC-SAXS-MALS-UV-visible absorption results for the activation of sGC**

| **Measurement** | **Inactive** | **1-NO** |
| --- | --- | --- |
| SAXS Molecular Weight (kDa) | ~140 | ~131 |
| MALS Molecular Weight (kDa) | 140.7 (±0.008%) | 138.4 (±0.014%) |
| Ratio 432/280 nm Absorbance | 0.765 | 0.319 |
| Radius of Gyration, R_g_ (Å) | 43.1 (±0.4) | 43.8 (±0.2) |
| Maximum Dimension, D_max_ (Å) | 133 | 142 |
| Porod Exponent, P_x_ | 4 | 4 |
| Inactive Conformation (%) | 100 | 72 |
| Partially Extended Conformation (%) | 0 | 28 |
| χ^2^-value of Fit | 1.44 | 1.51 |
